# The importance of endoscopic ultrasound fine-needle aspiration in the diagnosis of solid pseudopapillary tumor of the pancreas: two case reports

**DOI:** 10.1186/s13256-018-1585-3

**Published:** 2018-04-26

**Authors:** Diogo Turiani Hourneaux De Moura, Martin Coronel, Igor Braga Ribeiro, Galileu Ferreira Ayala Farias, Maria Auxiliadora Choez, Rodrigo Rocha, Marcello Pecoraro Toscano, Eduardo Guimarães Hourneaux De Moura

**Affiliations:** 1 Gastrointestinal Endoscopy Unit, Hospital das Clínicas da Faculdade de Medicina da Universidade de São Paulo, Av. Dr Enéas de Carvalho Aguiar, 225, 6o andar, bloco 3, Cerqueira Cezar, São Paulo, SP ZIP Code 05403-010 Brazil; 2 Pathology Unit, Hospital das Clínicas da Faculdade de Medicina da Universidade de São Paulo, Av. Dr Enéas de Carvalho Aguiar, 225, Andar, bloco, Cerqueira Cezar, São Paulo, SP ZIP Code 05403-010 Brazil

**Keywords:** Solid pseudopapillary tumor, Pancreatic malignancy, Frantz tumor, Endoscopic ultrasound fine-needle aspiration, Case series report

## Abstract

**Background:**

Solid pseudopapillary tumor of the pancreas, otherwise known as solid and cystic tumor or Frantz tumor, is an unusual form of pancreatic carcinoma, with unknown etiopathogenesis, that accounts for 0.2 to 2.7% of all pancreatic tumors. It is defined as an exocrine pancreatic neoplasia that mainly affects women between the second and third decade of life, and its management is not well defined. Endoscopic ultrasound offers a key anatomical advantage in accessing the pancreas and endoscopic ultrasound fine-needle aspiration has become the gold standard method for the diagnosis of pancreatic lesions.

**Case presentation:**

**Case 1:** A 31-year-old white Hispanic woman presented with epigastric pain for 5 months. An abdominal ultrasound revealed a single 2 cm nodule in the uncinate process of her pancreas. Endoscopic ultrasound showed a regular, well-defined solid lesion with alternating cystic areas at the uncinate process of her pancreas, measuring 1.7 × 1.4 cm; endoscopic ultrasound fine-needle aspiration was then performed with cytopathological analysis compatible with solid pseudopapillary tumor.

Body computed tomography confirmed the absence of metastases and she underwent conventional duodenopancreatectomy. However, she died 4 days after surgery due to postoperative surgical complications.

**Case 2:** A 35-year-old Hispanic woman presented with left upper quadrant abdominal pain for 3 months, associated with a palpable mass at this region. A computed tomography scan showed a solitary nodule in the pancreatic body. Endoscopic ultrasound showed a regular, well-defined, homogeneous lesion with small anechoic (cystic) areas, measuring 2 × 2 cm, in between the pancreatic body and neck. Endoscopic ultrasound fine-needle aspiration was performed and cytopathological analysis was suggestive of a pseudopapillary solid tumor. She underwent a body-tail laparoscopic pancreatectomy with splenectomy. Nine months after the diagnosis, she remains asymptomatic, continuing regular follow-up in the oncology out-patient clinic.

**Conclusions:**

Solid pseudopapillary tumor is a rare pancreatic malignancy. Endoscopic ultrasound fine-needle aspiration is the gold standard method to characterize and diagnose this type of pancreatic lesion, making this an invaluable tool to help guide clinical management and improve the preoperative diagnostic yield.

## Background

Solid pseudopapillary tumor (SPT) of the pancreas, otherwise known as solid and cystic tumor or Frantz tumor, is a rare but characteristic neoplasm, with unknown etiopathogenesis, accounting for 0.2 to 2.7% of all pancreatic tumors and less than 5% of pancreatic cystic tumors [1–3]. It is defined as an exocrine pancreatic neoplasia that mainly affects women between the second and third decade of life and is rarely seen in men or children [2]. When present in men, it has greater malignant potential with a worse prognosis [4]. It accounts for approximately 8 to 16% of pancreatic tumors in children [5].

Symptoms of SPT depend on the location and size of the tumor but usually are nonspecific, with abdominal pain being the most common in approximately one-third of patients [6]. Several imaging techniques can be used to diagnose pancreatic masses, such as abdominal ultrasound (US), computed tomography (CT), magnetic resonance imaging (MRI), and endoscopic ultrasound (EUS). EUS has assumed a very important role in the diagnosis of pancreatic lesions, providing a better evaluation of the morphologic characteristics of the lesions and the possibility of guiding fine-needle aspiration (FNA) punctures for tissue sampling with a low risk of complications and increased diagnostic accuracy [7]. SPT can present as a solid, cystic, or mixed lesion [8, 9].

The treatment of choice is a complete curative surgical resection of the lesion. The long-term prognosis is excellent, since it has a generally indolent behavior and a low degree of malignancy [5].

Here we report two cases of SPT diagnosed by preoperative EUS-FNA, presenting distinct clinical outcomes after a proper surgical approach.

## Case presentation

### Case 1

**Fig. 1:**
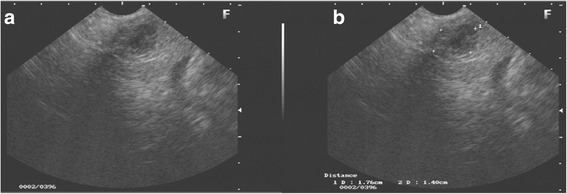
Endoscopic ultrasound view of the solid cystic lesion in the pancreas. **a** Rounded lesion, with well-defined contours, sharp and precise borders, with solid and cystic areas. **b** Solid lesion with cystic components measuring 1.7 × 1.4 cm

A 31-year-old white Hispanic woman, who did not smoke tobacco or consume alcohol, presented with a 5-month history of epigastric pain. She did not present with any other symptoms. An abdominal US revealed a 2 cm, single nodule in the uncinate process of her pancreas. EUS showed a well-defined hypoechoic solid lesion with regular, clear, and precise margins with alternating cystic areas measuring 1.7 × 1.4 cm, located in the uncinate process of her pancreas; the lesion had no communication with her main pancreatic duct (Fig. 1a, b). EUS-FNA was performed with a 22 gauge needle (Expect™ Slimline; Boston Scientific) obtaining a representative tissue sample without complications. A cytopathological study showed single cells, small loose clusters, and scattered intact papillary structures with fibrovascular components, finely granular cytoplasm, and nuclei with fine chromatin, consistent with SPT of the pancreas (Fig. 3a).

A body CT scan was performed, excluding metastatic disease. She underwent conventional duodenopancreatectomy (Whipple procedure). However, she evolved with postoperative sepsis and died on the fourth day after surgery.

### Case 2

**Fig. 2:**
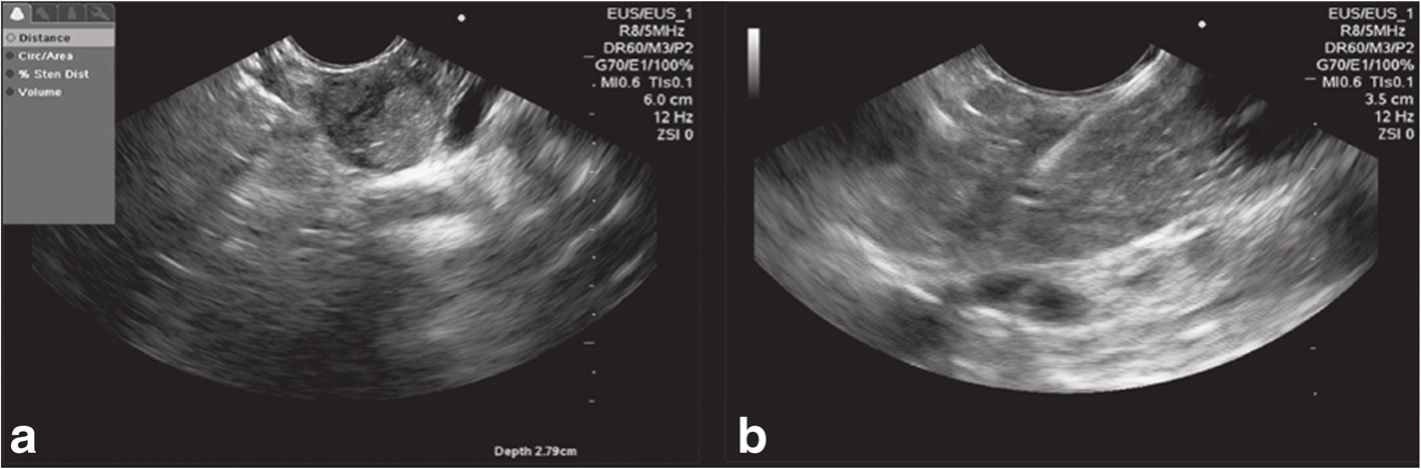
Endoscopic ultrasound view of the solid lesion with cystic areas in the pancreas. **a** Endoscopic ultrasound showed a regular, well-defined, homogeneous lesion with small anechoic (cystic) areas, measuring 2 × 2 cm, in the pancreatic body to neck transition. **b** Endoscopic ultrasound fine-needle aspiration with a 22 gauge needle

**Fig. 3:**
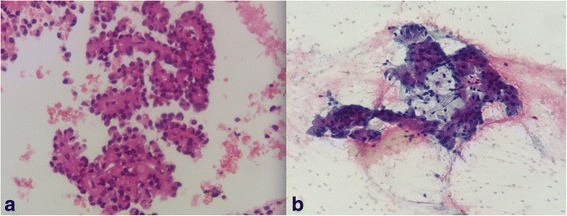
Histopathologic plates analysis of solid pseudopapillary tumor. **a** Cellular, single cells, small loose clusters, and scattered intact papillary structures with delicate fibrovascular cores, finely granular cytoplasm, and nuclei with fine chromatin. **b** Well-differentiated epithelial neoplasm, with papillary structures

A 35-year-old Hispanic woman presented with left upper quadrant abdominal pain for 3 months, associated with a palpable mass at this region. An abdominal CT scan showed a solitary nodule in the pancreas body. EUS showed a regular, well-defined, homogeneous lesion with small anechoic (cystic) areas, measuring 2 × 2 cm, localized between the body and the neck of the pancreas (Fig. 2a). EUS-FNA was then performed with a 22 gauge needle (Fig. 2b) obtaining a representative tissue sample without complications. The cytopathological analysis (four slides and one cell block) showed aspects compatible with SPT of the pancreas (Fig. 3b). An immunohistochemistry study was positive for Ki-67 antigen (with low mitotic index of 10%) and positive for beta-catenin, CD56, chromogranin, focal receptor of progesterone, and focal synaptophysin. She underwent a body-tail laparoscopic pancreatectomy with splenectomy. Nine months after the diagnosis, she remains asymptomatic and follow-up continues in the oncology out-patient clinic.

## Discussion

SPT of the pancreas was first described by Frantz in 1959 [5]. It is a very rare type of pancreatic neoplasm with a low rate of progression to malignancy [10]. Under 2800 cases have been reported until the year 2012 [11], with the largest single institution case series of 37 patients who underwent surgical resection of SPT [12]. It affects mainly young women (approximately 90% of cases), but can affect men and women of any age group [4]. The most commonly affected areas are the body and tail of the pancreas, corresponding to approximately 60% of the diagnosed cases. Unlike most reported cases, our patients presented lesions at the uncinate process and at the transition between the body and neck of the pancreas [11].

The symptoms of SPT are usually nonspecific, with abdominal pain being the most common, accounting for approximately 37.6% of the cases. Other signs and symptoms such as jaundice, abdominal fullness, anorexia, nausea, vomiting, and weight loss may also be present. Approximately one-third of the patients are asymptomatic [6]. As reported in the literature, our patients presented with nonspecific symptoms. With the recent advances of radiologic diagnostic imaging methods, SPT has been increasingly diagnosed in general clinical practice. US, CT, MRI, and EUS are the most widely used methods for pancreatic masses evaluation. Over the years, EUS-FNA has become the gold standard method for the diagnosis of pancreatic solid masses [13]. The sensitivity and specificity of EUS-FNA for the diagnosis of pancreatic neoplasms ranges from 80 to 90% and from 85 to 96%, respectively [13]. EUS commonly identifies SPT as a well circumscribed, solid, hypoechogenic, heterogeneous tumor with cystic components and calcifications [14].

In our two cases, EUS showed solid hypoechoic lesion with cystic components with regular contours but it was not heterogeneous. Since EUS-FNA is considered the gold standard for pancreatic lesions, EUS-FNA was performed with histopathological diagnostic confirmation of SPT.

The diagnosis of SPT with EUS-FNA has been increasing in popularity as shown in a case series of 34 patients [15], where EUS-FNA improved the preoperative diagnostic yield of SPT, correctly identifying 82% of patients when compared to CT scan findings (23.5%) and EUS (41.2%). These findings suggest that patients who do not have a clear diagnosis of an SPT using only image studies may benefit from EUS-FNA [15]. The operating characteristics of EUS-FNA for diagnostic solid pancreatic masses were: sensitivity 95% (95% CI, 93.2–95.4), specificity 92% (95% CI, 86.6–95.7), positive predictive value 98% (95% CI, 97–99), and negative predictive value 80% (95% CI, 74.9–82.7). The overall accuracy of EUS-FNA was 94.1% (95% CI, 92.0–94).

Complications related to EUS-FNA are only reported in approximately 1% of patients. The most common complication is acute pancreatitis; other reported complications are abdominal pain, fever, vomiting, and bleeding [7]. Both of our cases presented no complications related to the EUS-FNA procedure.

An anatomopathological study revealed pseudopapillary areas with fibrovascular stems or rosette-like structures secondary to the low cohesion of neoplastic cells [16]. An immunohistochemical study stained positive for beta-catenin, vimentin, progesterone receptor, CD56, neuron-specific coil, CD10, cyclin D1, E-membranous, and E-cadherin [6]. Unlike other pancreatic tumors, the most sensitive specific marker for SPT is the abnormal nuclear expression of beta-catenin [17]. The Ki-67 index has been suggested as an indicator of malignant potential and poor prognosis in SPT. A low Ki-67 index (≤ 5%) indicates slow tumor growth [18]. Microscopic evaluation of the two reported cases revealed intact papillary structures with fibrovascular stems. In the second case, diagnostic confirmation was still necessary through an immunohistochemical study, confirming the suspected diagnosis of SPT through the expression of beta-catenin. Immunohistochemistry also showed a low mitotic Ki-67 index, suggesting good prognosis.

Surgical treatment of SPT offers an excellent overall survival rate, greater than 95% [6]. The surgical procedure to be performed will depend on the topography and level of tumor invasion [19]. After diagnostic confirmation by EUS-FNA, our two patients underwent different surgical procedures according to lesion topography. Patient 1 underwent duodenopancreatectomy, while patient 2 underwent a body-tail pancreatectomy with splenectomy. The outcomes of our two case reports were different, with one patient presenting a favorable evolution, while the other patient died due to postoperative complications. The different evolutions of the cases may justify the view that more aggressive surgery (duodenopancreatectomy) could have been performed on the patient with uncinated pancreatic process neoplastic invasion. The reported mortality rate related to postoperative complications after SPT resection is only 1% [5].

Neither of our patients had neoplastic metastases, although it is known that despite the low malignant potential, up to 15% of cases of SPT develop distant metastases [20]. The most common sites of metastasis are the liver and lymph nodes. Tumor recurrence may occur in approximately 6% of cases, requiring rigorous follow-up [5].

In this context, EUS-FNA is a very important method that presents excellent diagnostic rates and provides material for cytological and histological evaluation of SPT.

## Conclusions

SPT is a rare pancreatic neoplasia. Anatomopathological study is necessary for treatment and prognosis evaluation. EUS-FNA is the gold standard method to characterize and diagnose these type of pancreatic lesions, making this an invaluable tool to help guide clinical management, decide a patient’s surgical candidacy, and improve the preoperative diagnostic yield.

## Abbreviations

CT: Computed tomography
EUS: Endoscopic ultrasound
FNA: Fine-needle aspiration
MRI: Magnetic resonance imaging
SPT: Solid pseudopapillary tumor
US: Ultrasound

## References

[1] Branco C, Vilaça S, Falcão J. Solid pseudopapillary neoplasm. Case report of a rare pancreatic tumor. Int J Surg Case Rep. 2017;33:148–50. 10.1016/j.ijscr.2017.02.049 PMC5358903 28327418

[2] Słowik-Moczydłowska Z, Gogolewski M, Yaqoub S. Solid pseudopapillary tumor of the pancreas (Frantz’s tumor): two case reports and a review of the literature. J Med Case Rep. 2015;9:268. 10.1186/s13256-015-0752-z PMC4653910 26585230

[3] Canzonieri V, Berretta M, Buonadonna A, Libra M, Vasquez E, Barbagallo E, Bearz A, Berretta S. Solid pseudopapillary tumour of the pancreas. Lancet Oncol. 2003;4(4):255–6. 10.1016/s1470-2045(03)01038-6 12681270

[4] Lin MYC, Stabile BE. Solid pseudopapillary neoplasm of the pancreas: a rare and atypically aggressive disease among male patients. Am Surg. 2010;76(10):1075–8. 21105613

[5] Papavramidis T, Papavramidis S. Solid pseudopapillary tumors of the pancreas: review of 718 patients reported in the English literature. J Am Coll Surg. 2005;2:965–72. 10.1016/j.jamcollsurg.2005.02.011 15922212

[6] Yu PF, Hu ZH, Wang XB, Guo JM, Cheng XD, Zhang YL, et al. Solid pseudo papillary tumor of the pancreas: a review of 553 cases in Chinese literature. World J Gastroenterol. 2010;16(10):1209–14. 10.3748/wjg.v16.i10.1209 PMC2839172 20222163

[7] Eloubeidi MA, Varadarajulu S, Desai S, Shirley R, Heslin MJ, Mehra M, et al. A prospective evaluation of an algorithm incorporating routine preoperative endoscopic ultrasound-guided fine needle aspiration in suspected pancreatic cancer. J Gastrointest Surg. 2007;11(7):813–9. 10.1007/s11605-007-0151-x 17440790

[8] Anil G, Zhang J, Al Hamar NE, Nga ME. Solid pseudopapillary neoplasm of the pancreas: CT imaging features and radiologic-pathologic correlation. DiagnIntervRadiol. 2017;23(2):94–9. 10.5152/dir.2016.16104 PMC5338587 28089954

[9] Aso A, Ihara E, Nakamura K, Sudovykh I, Ito T, Nakamura M, Ikeda T, Takizawa N, Oda Y, Shimizu S. Solid Pseudopapillary Neoplasm of the Pancreas in Young Male Patients: Three Case Reports. Case Rep Gastrointest Med. 2017;2017:9071678. 10.1155/2017/9071678 PMC5306977 28255475

[10] Frantz VK. Atlas of tumor pathology, section VII. Washington DC: Armed Forces Institute of Pathology; 1959. p. 28. Tumors of the pancreas. [fascicles 27]

[11] Law JK, Ahmed A, Singh VK, et al. A systematic review of solid-pseudopapillary neoplasms: are these rare lesions? Pancreas. 2014;43(3):331–7. 10.1097/MPA.0000000000000061 PMC4888067 24622060

[12] Reddy S, Cameron JL, Scudiere J, Hruban RH, Fishman EK, Ahuja N, Pawlik TM, Edil BH, Schulick RD, Wolfgang CL. Surgical management of solid-pseudopapillary neoplasms of the pancreas (Franz or Hamoudi tumors): a large single-institutional series. J Am Coll Surg. 2009;208(5):950–7. 10.1016/j.jamcollsurg.2009.01.044 PMC3109868 19476869

[13] Banafea O, Mghanga FP, Zhao J, Zhao R, Zhu L. Endoscopic ultrasonography with fine-needle aspiration for histological diagnosis of solid pancreatic masses: a meta-analysis of diagnostic accuracy studies. BMCGastroenterol. 2016;16:108. 10.1186/s12876-016-0519-z PMC5007683 27580856

[14] Jung WS, Kim JK, Yu JS, Kim JH, Cho ES, Chung JJ. Comparison of abdominal ultrasonographic findings with endoscopic ultrasonographic findings of solid pseudopapillary neoplasms of the pancreas. Ultrasound Q. 2014;30(3):173–8. 10.1097/RUQ.0000000000000093 25148485

[15] Law K, Stoita A, Weaver W, Gleeson FC, Dries AM, Blackford A, et al. Endoscopic ultrasound-guided fine needle aspiration improves the pre-operative diagnostic yield of solid-pseudopapillary neoplasm of the pancreas: an international multicenter case series (with video). SurgEndosc. 2014;9:2592–8. 10.1007/s00464-014-3508-8 24718662

[16] Pettinato G, Manivel JC, Ravetto C, et al. Papillary cystic tumor of the pancreas. A clinicopathologic study of 20 cases with cytologic, immunohistochemical, ultrastructural, and flow cytometric observations, and a review of the literature. Am J ClinPathol. 1992;98:478–88. 10.1093/ajcp/98.5.478 1283055

[17] Tanaka Y, Kato K, Notohara K, et al. Frequent beta-catenin mutation and cytoplasmic/nuclear accumulation in pancreatic solid-pseudopapillary neoplasm. Cancer Res. 2001;61:8401–4. 11731417

[18] Yu P, Cheng X, Du Y. Solid pseudopapillary neoplasms of the pancreas: a 19-year multicenter experience in China. J Gastrointest Surg. 2015;19:1433–40. 10.1007/s11605-015-2862-8 26001371

[19] Cheng-Hong P, Dong-Feng C, Guang-Wen Z, Yang M. The solid pseudopapillary tumor of pancreas: the clinical characteristics and surgical treatment. J Surg Res. 2006;131:276–82. 10.1016/j.jss.2005.11.585 16457845

[20] Tang LH, Aydin H, Brennan MF, Klimstra DS. Clinically aggressive solid pseudopapillary tumors of the pancreas: a report of two cases with components of undifferentiated carcinoma and a comparative clinicopathologic analysis of 34 conventional cases. Am J Surg Pathol. 2005;29:512–9. 10.1097/01.pas.0000155159.28530.88 15767807

